# Insulinotropic Activity of Standardized Methanolic Extracts of* Ficus deltoidea* from Seven Varieties

**DOI:** 10.1155/2018/3769874

**Published:** 2018-06-26

**Authors:** Nurshieren Yahaya, Nur Sumirah Mohd Dom, Zainah Adam, Muhajir Hamid

**Affiliations:** ^1^Department of Microbiology, Faculty of Biotechnology and Biomolecular Sciences, Universiti Putra Malaysia, 43400 Serdang, Selangor, Malaysia; ^2^Medical Technology Division, Malaysian Nuclear Agency, 43000 Kajang, Selangor, Malaysia

## Abstract

*Ficus deltoidea *is a traditional medicinal plant that has been proven to show antidiabetic effects. This study focus is to assess the insulin secretion activity of* Ficus deltoidea* standardized methanolic extracts from seven independent varieties and mechanisms that underlie the insulin secretion action of the extracts. The cytotoxicity of* Ficus deltoidea *extracts was tested using viability assay. The insulin secretion assay was carried out by treating clonal BRIN BD11 cell line with standardized methanolic* Ficus deltoidea* extracts or glybenclamide. The clonal BRIN BD11 cell was also treated with insulin agonist and antagonist to elucidate the insulin secretion mechanism. Only the viability percentage for* Ficus deltoidea *var.* kunstleri *and* intermedia *was identified to be toxic at 500 and 1000 *μ*g/ml (*P*<0.001). The insulin secretion for* Ficus deltoidea *var.* deltoidea, angustifolia*, and* motleyana* was dose-dependent; further evaluation suggested that* Ficus deltoidea* var.* trengganuensis *was involved in K_ATP_-independent pathway. This study suggests that standardized methanolic extracts of* Ficus deltoidea *varieties have an insulinotropic effect on clonal BRIN BD11 cell line and can be utilized as a modern candidate of antidiabetic agents targeting the escalation for insulin secretion from pancreatic beta cells.

## 1. Introduction

Diabetes refers to a metabolic disorder that can potentially lead to death in the case the disorder is not treated and managed properly. Diabetes can be diagnosed into types, namely, types 1 and 2. The condition for type 1 diabetes includes impaired beta pancreatic cell leading to failure of insulin secretion production, and the condition of the patient can be treated by means of injection of insulin. The other type of diabetes indication includes insulin-targeting cells are insensitive to insulin even though insulin is present in body [1]. In addition, 422 million adults were diagnosed with this metabolic disease in 2014 on a global scale, compared to only 108 million in 1980 [2]. This rise in the number of affected people is worrisome, as diabetes can cause complications and eventually fatality, thus having a harmful impact on the world population and economic growth in general. Therefore, techniques to manage diabetes by targeting the molecular mechanisms of action related to diabetes mellitus have become increasingly important to researchers and scientists during the past few years.

Even though there exist various synthetic drugs such as metformin available in the market as a means to treat diabetes, there are adverse effects from consuming such drugs. For example, metformin can potentially lead to lactic acidosis, which causes nausea and vomiting [3]. Therefore, targeting molecular mechanisms for diabetes using phytopharmaceutical agents from herbal medicine has become a recent trend in competing to find the best possible cure for this metabolic disorder.* Ficus deltoidea *is a medicinal plant that is found in several areas in peninsular Malaysia and Sarawak. There are 25 different varieties reported by Berg, seven of which have been taxonomically researched, namely,* Ficus deltoidea *variety* deltoidea, angustifolia, kunstleri, trengganuensis, intermedia, motleyana, *and* bilobata* [4]. It has been reported that the leaves of this plant have the potential to treat diabetes, as they can lower blood glucose levels [5, 6]. In this study, the insulin secretion activity of* Ficus deltoidea* standardized methanolic extracts from seven varieties and the mechanisms that underlie the insulin secretion action of the extracts were evaluated using BRIN BD11 pancreatic beta cell line. The cytotoxic activity of the extracts against such cells was also evaluated.

## 2. Materials and Methods

### 2.1. Plant Extracts Preparation

The standardized methanolic extracts of* Ficus deltoidea* varieties were retrieved from Professor Dr. Nor Hadiani Ismail Laboratory, Universiti Teknologi MARA (UiTM), Puncak Alam campus, and then stored at -20°C prior to the experiments.

### 2.2. Cell Culture

BRIN BD11 cell line was cultured with RPMI medium supplied with 10 % (v/v) FBS and 1% (v/v) Penicillin Streptomycin antibiotics. The cells were incubated at 37°C and humidified with 5% CO_2_.

### 2.3. Viability Assay

Viability assay was investigated using previously established methods [7, 8] with slight modifications. Confluence cells in 75 cm^3^ culture flasks were seeded with 1.5 x 10^5^ cells/ml stocks into 96 well plates. 100 *μ*l of cells were added with 100 *μ*l of complete media and left to attach for 12 hours at 37°C. The media were aspirated on the following day, and 100 *μ*l of the test substances at 20, 100, 200, 1000, and 2000 *μ*g/ml with 100 *μ*l new media was added which include untreated control,* Ficus deltoidea *extracts, and glybenclamide. After incubation of 72 hours at 37°C, 20 *μ*l of MTT solution (5 mg/ml) was added to each well and incubated for 4 hours at 37°C. Next, the media were gently discarded, and 100 *μ*l of DMSO was added. Then, the absorbance was measured at 570 nm using a Multimode Plate Reader (PerkinElmer, USA). The assay was repeated for three times. The viability percentage was calculated using the following formula.

(1) $$\begin{array}{c} \%\text{ Cell viability}=\frac{\text{Absorbance of samples}}{\text{Absorbance of control}}\text{ x }100 \end{array}$$

### 2.4. Insulin Secretion Assay

The effect of standardized methanolic* Ficus deltoidea* varieties on insulin secretion was evaluated using clonal pancreatic beta cell line. BRIN BD11 were seeded at 2.5 x 10^5^ cells/ml in 12 well plate and incubated for 24 hours at 37°C. Next, the cells were washed thrice with buffer and preincubated with Krebs-Ringer bicarbonate buffer for 40 minutes and continued for 60 minutes of incubation with the extracts (10-1000 *μ*g/ml) or glybenclamide (10-2000 *μ*g/ml). Ultrasensitive Rat Insulin ELISA kit (Mercodia AB, Sweden) was used to determine insulin concentration [5].

### 2.5. Elucidation of Insulin Secretion Mechanism Assay

The standardized methanolic* Ficus deltoidea *extracts concentrations that demonstrated significant effect of insulin secretions were further evaluated. The cells were seeded at a concentration of 2.5 x 10^5^ cells/well in 12-wells plates and incubated for 24 hours at 37°C. Then, the cells were washed thrice with buffer and preincubated with Krebs-Ringer bicarbonate buffer (KRB) for 40 minutes. Next, incubation was carried out for 60 minutes with standardized methanolic* Ficus deltoidea *extract with and without 100 *μ*M of isobutylmethylxanthine (IBMX), 200 *μ*M of tolbutamide, 300 *μ*M of diazoxide, and 30 mM of KCl. Ultrasensitive Rat Insulin ELISA kit (Mercodia AB, Sweden) was used to determine insulin concentration [5, 9].

### 2.6. Statistical Analysis

The data was expressed as mean ± SD and then analysed using one-way ANOVA, followed by Tukey's post hoc test in GraphPad Prism version 6.

## 3. Results

### 3.1. Cell Viability Effects of Standardized Methanolic Ficus deltoidea Varieties

The effects of standardized methanolic extract* Ficus deltoidea* varieties and glybenclamide towards pancreatic beta cell line are presented in Table 1.* Ficus deltoidea* var.* kunstleri* and* intermedia* have less than 50% viability starting from a concentration of 500 *μ*g/ml, while* Ficus deltoidea* var.* deltoidea* and* bilobata* have less than 50% cell viability at 1000 *μ*g/ml. Glybenclamide showed less than 50% cell viability starting from 1000 *μ*M.

### 3.2. Insulin Secretion Activity of Standardized Methanolic Ficus deltoidea Varieties

**Figure 1:**
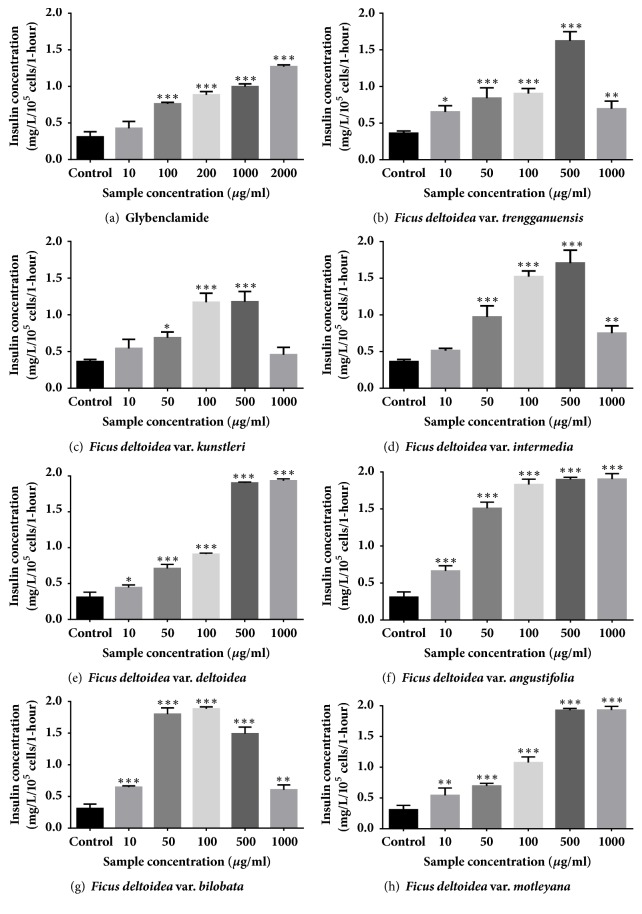
Effect of standardized methanolic* Ficus deltoidea *varieties and glybenclamide in insulin secretion on BRIN BD11 pancreatic beta cells for 1-hour incubation. Results are exhibited as mean ± SD (n=4) of insulin concentration. ^*∗*^*P*<0.05, ^*∗∗*^*P*<0.01, and ^*∗∗∗*^*P*<0.001 compared to control.

The effects on insulin secretion by* Ficus deltoidea *varieties and glybenclamide in BRIN BD11 pancreatic beta cell are shown in Figure 1. Glybenclamide demonstrated a significant stepwise excitatory action on insulin secretion at concentrations of 100, 200, 1000, and 2000 *μ*M, which increased at 2.46 (*P*<0.001), 2.86 (*P*<0.001), 3.22 (*P*<0.001), and 4.10 (*P*<0.001) fold, respectively.* Ficus deltoidea* var.* trengganuensis*, var.* kunstleri,* and* intermedia* at 500 ug/ml showed the highest insulin secretion, which evoked 8.61 (*P*<0.001), 5.62 (*P*<0.001), and 9.11 (*P*<0.001) fold, respectively. In addition,* Ficus deltoidea *var.* bilobata *showed the highest insulin secretion at 100 *μ*g/ml, which evoked 6.07 (*P*<0.001) fold.* Ficus deltoidea *var.* deltoidea, angustifolia, *and* motleyana *showed a significant stepwise stimulatory effect of given concentrations.

### 3.3. Elucidation of Insulin Secretion Mechanism

**Figure 2:**
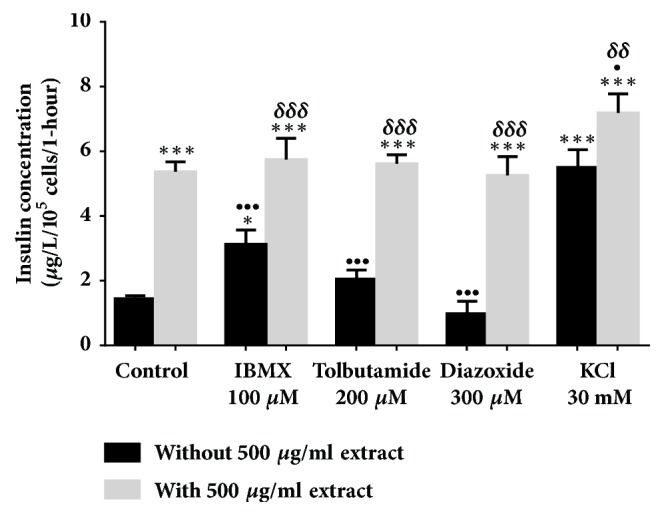
Effect of four modulators on BRIN BD 11 treated with 500 *μ*g/ml standardized methanolic extract of* Ficus deltoidea* var.* trengganuensis*. Values represent mean ± standard deviation (n=4) of insulin concentration. ^*∗*^*P*<0.05, ^*∗∗∗*^*P*<0.001 compared to control without extract, while ^•^*P*<0.05, ^•••^*P*<0.001 compared to control with extract, and ^*δδ*^*P*<0.01, ^*δδδ*^*P*<0.001, compared to modulator without extract in respective group.

**Figure 3:**
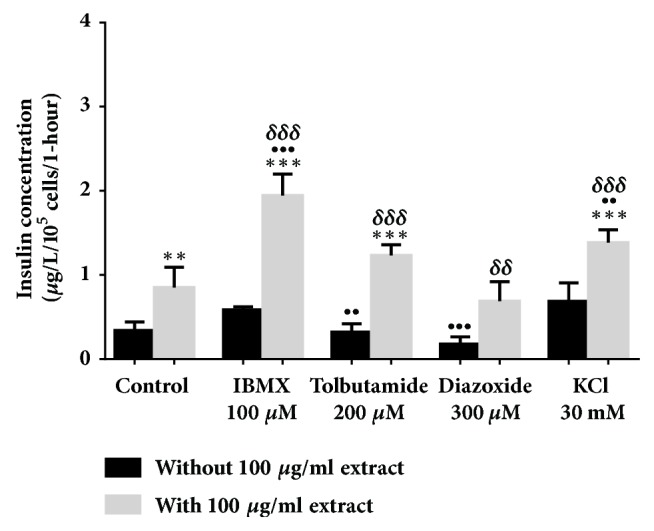
Effect of four modulators on BRIN BD 11 treated with 100 *μ*g/ml standardized methanolic extract of* Ficus deltoidea *var.* kunstleri*. Values represent mean ± standard deviation (n=4) of insulin concentration. ^*∗∗*^*P*<0.01,^*∗∗∗*^*P*<0.001 compared to control without 100 *μ*g/ml extract, while ^••^*P*<0.01, ^•••^*P*<0.001 compared to control with extract, and ^*δδδ*^*P*<0.001 compared to modulator without extract in respective group.

**Figure 4:**
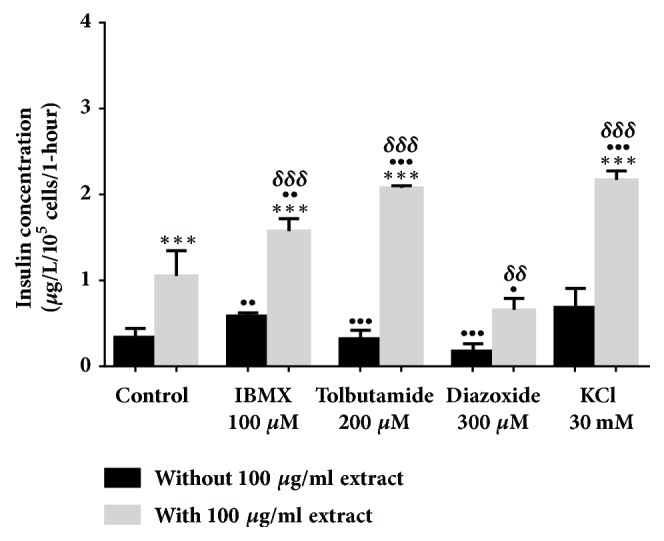
Effect of four modulators on BRIN BD 11 treated with 100 *μ*g/ml standardized methanolic* Ficus deltoidea *var.* intermedia*. Values represent mean ± standard deviation (n=4) of insulin concentration. ^*∗∗∗*^*P*<0.001 compared to control without extract, while ^•^*P*<0.05, ^••^*P*<0.01, ^•••^*P*<0.001 compared to control with extract, and ^*δ*^*P*<0.05, ^*δδ*^*P*<0.01, and ^*δδδ*^*P*<0.001 compared to modulator without extract in respective group.

**Figure 5:**
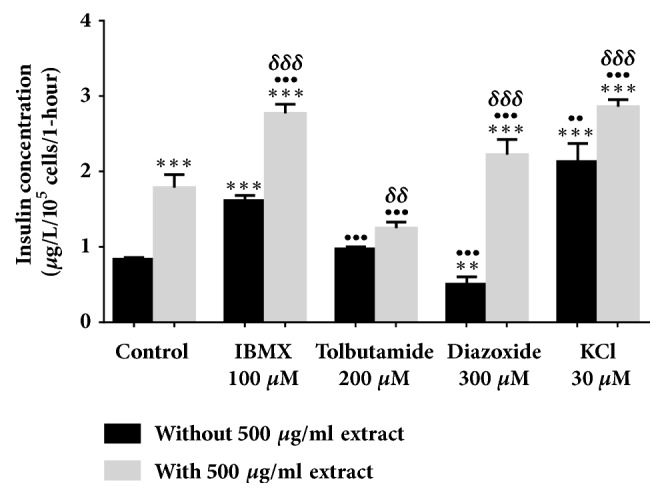
Effect of four modulators on BRIN BD 11 treated with 500 *μ*g/ml standardized methanolic* Ficus deltoidea *var.* deltoidea*. Values represent mean ± standard deviation (n=4) of insulin concentration. ^*∗∗*^*P*<0.01, ^*∗∗∗*^*P*<0.001 compared to control without extract, while ^••^*P*<0.01, ^•••^*P*<0.001 compared to control with extract, and ^*δδ*^*P*<0.01, ^*δδδ*^*P*<0.001 compared to modulator without extract in respective group.

All evaluations were carried out in a 2 mM glucose concentration. In Figure 2, 500 *μ*g/ml* Ficus deltoidea *var.* trengganuensis* stimulates insulin secretion at 3.73 fold (*P*<0.001). Combinations of 500 *μ*g/ml* Ficus deltoidea *var.* trengganuensis* with the modulators intrigued insulin secretion with magnitudes of potentiation of 1.84 fold (*P*<0.001), 2.74 fold (*P*<0.001), 5.37 fold (*P*<0.001), and 1.31 fold (*P*<0.01), for 100 *μ*M of IBMX, 200 *μ*M of tolbutamide, 300 *μ*M of diazoxide, and 30 mM of KCl, respectively. Only a combination of the extract with 30 mM of KCl increased insulin secretion with 1.34 fold (*P*<0.05) when compared to the extract alone. In Figure 3, the escalation of insulin secretion by 100 *μ*g/ml* Ficus deltoidea *var.* kunstleri* was shown at 2.50 fold (*P*<0.01). The insulin-releasing potentiation when combined with modulators was 3.18 fold (*P*<0.001), 3.84 fold (*P*<0.001), 3.87 fold (*P*<0.01), and 2.02 fold (*P*<0.001), for 100 *μ*M of IBMX, 200 *μ*M of tolbutamide, 300 *μ*M of diazoxide, and 30 mM of KCl, respectively. Combinations of 100 *μ*g/ml of standardized* Ficus deltoidea *var.* kunstleri *methanolic extract with 100 *μ*M of IBMX and 30 mM of KCl escalated the insulin-releasing effect. The increase in insulin secretion was 2.29 fold (*P*<0.001) and 1.63 fold (*P*<0.01), respectively.  Figure 4 shows the insulin secretion potential of 100 *μ*g/ml* Ficus deltoidea *var.* intermedia*, which potentiated insulin secretion by 3.10 fold (*P*<0.001). The insulin secretion magnitudes, when combined with the modulators, were 2.69 fold (*P*<0.001), 6.45 fold (*P*<0.001), 3.69 fold (*P*<0.01), and 3.16 fold (*P*<0.001). The magnitude of the insulin secretions was 1.50 fold (*P*<0.01), 1.98 fold (*P*<0.001), and 2.07 fold (*P*<0.001), compared to the untreated control for 100 *μ*M of IBMX, 200 *μ*M tolbutamide, and 30 mM of KCl, respectively. The magnitude of the inhibition was 0.63 fold (*P*<0.05) for 300 *μ*M of diazoxide. In Figure 5,* Ficus deltoidea *var.* deltoidea* showed an escalation of basal insulin secretion of 2.13 fold (*P*<0.001) when incubated with 500 *μ*g/ml extract. The combinations of modulators with 500 *μ*g/ml* Ficus deltoidea *var.* deltoidea* extract showed a magnitude of insulin secretion escalation of 1.72 fold (*P*<0.001), 1.28 fold (*P*<0.01), 4.40 fold (*P*<0.001), and 1.34 fold (*P*<0.001) for 100 *μ*M of IBMX, 200*μ*M tolbutamide, 300 *μ*M of diazoxide and 30 mM of KCl, respectively. The magnitude of the potentiation was 1.03 fold (*P*<0.001) for the combination of extracts with 30 mM of KCl, when compared to untreated control. The magnitude of inhibition was 0.80 fold (*P*<0.001) for the combination of extracts with 300 *μ*M of diazoxide when compared to untreated control. The combination of 500 *μ*g/ml of standardized* Ficus deltoidea *var.* deltoidea *methanolic extract with 100 *μ*M of IBMX, 300 *μ*M of diazoxide, and 30 mM of KCl increased the insulin triggering effect when compared to control. The magnitudes of escalation were 1.55 fold (*P*<0.001), 1.24 fold (*P*<0.001), and 1.60 fold (*P*<0.001), respectively.

**Figure 6:**
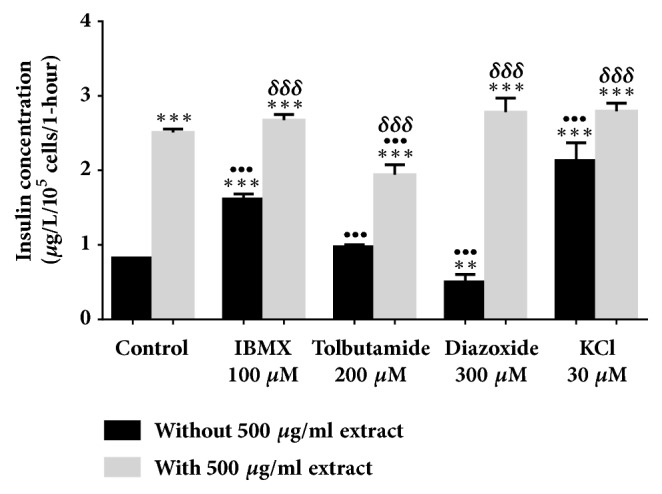
Effect of four modulators on BRIN BD 11 treated with 500 *μ*g/ml standardized methanolic* Ficus deltoidea *var.* angustifolia*. Values represent mean ± standard deviation (n=4) of insulin concentration. ^*∗∗*^*P*<0.01, ^*∗∗∗*^*P*<0.001 compared to control without extract, while ^•••^*P*<0.001 compared to control with extract and ^*δδδ*^*P*<0.001 compared to modulator without extract in respective group.

**Figure 7:**
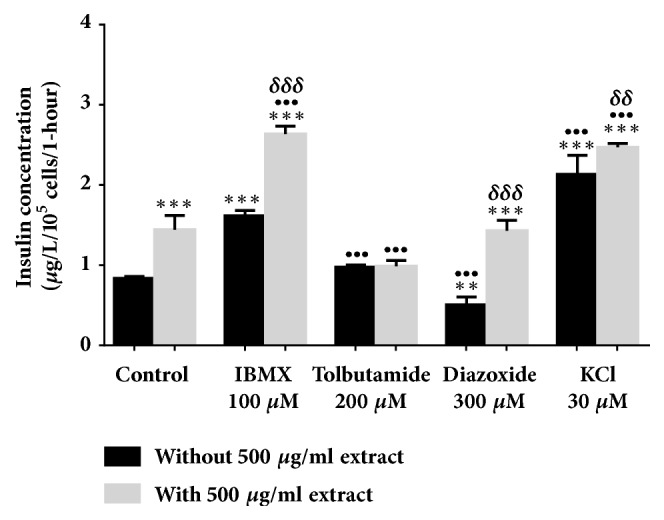
Effect of four modulators on BRIN BD 11 treated with 500 *μ*g/ml standardized methanolic* Ficus deltoidea *var.* bilobata*. Values represent mean ± standard deviation (n=4) of insulin concentration. ^*∗∗*^*P*<0.01, ^*∗∗∗*^*P*<0.001 compared to control without extract, while ^•••^*P*<0.001 compared to control with extract, and ^*δδ*^*P*<0.01, ^*δδδ*^*P*<0.001 compared to modulator without extract in respective group.

**Figure 8:**
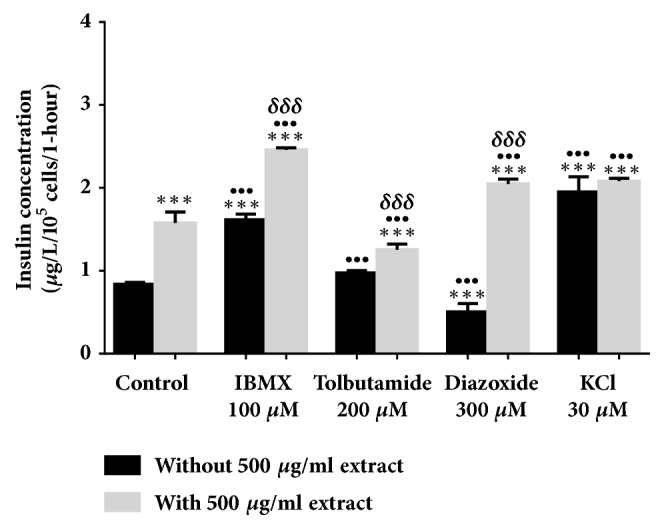
Effect of four modulators on BRIN BD 11 treated with 500 *μ*g/ml standardized methanolic* Ficus deltoidea *var.* motleyana*. Values represent mean ± standard deviation (n=4) of insulin concentration. ^*∗∗∗*^*P*<0.001 compared to control without extract, while ^•••^*P*<0.001 compared to control with extract, and ^*δδδ*^*P*<0.001 compared to modulator without extract in respective group.

In Figure 6, 500 *μ*g/ml* Ficus deltoidea *var.* angustifolia* increased basal insulin secretion at 2.99 fold (*P*<0.001). The combination of 500 *μ*g/ml* Ficus deltoidea *var.* angustifolia *with modulators showed a magnitude of insulin secretion of 1.13 fold (*P*<0.001), 1.33 fold (*P*<0.001), 5.51 fold (*P*<0.001), and 1.31 fold (*P*<0.001) when compared to 100 *μ*M of IBMX, 200 *μ*M tolbutamide, 300 *μ*M of diazoxide, and 30 mM of KCl, respectively. In Figure 7, 500 *μ*g/ml* Ficus deltoidea *var.* bilobata* triggered insulin secretion at 1.72 fold (*P*<0.001). The combination of 500 *μ*g/ml* Ficus deltoidea *var.* bilobata *with modulators yielded a magnitude of insulin secretion escalation at 1.63 fold (*P*<0.001), 2.95 fold (*P*<0.001), and 1.16 fold (*P*<0.01), compared to 100 *μ*M IBMX, 300 *μ*M of diazoxide, and 30 mM of KCl, respectively. The combination of 500 *μ*g/ml* Ficus deltoidea *var.* bilobata* with 100 *μ*M of IBMX and 30 mM of KCl showed a magnitude of insulin potentiation of 1.83 fold (*P*<0.001) and 1.72 fold (*P*<0.001) when compared to the extract alone, while 200 *μ*M tolbutamide showed a magnitude of insulin secretion inhibition by having 0.69 fold (*P*<0.001) when compared to the control with 500 *μ*g/ml* Ficus deltoidea *var.* bilobata* extract. Finally, in Figure 8, 500 *μ*g/ml* Ficus deltoidea *var.* motleyana *showed an escalation of 1.88 fold (*P*<0.001) when compared to untreated control. The combination of 500 *μ*g/ml* Ficus deltoidea *var.* motleyana* with modulators showed a magnitude of insulin secretion by 1.52 fold (*P*<0.001), 1.29 fold (*P*<0.001), and 4.05 fold (*P*<0.001) for 100 *μ*M of IBMX, 200 *μ*M tolbutamide, and 300 *μ*M of diazoxide, respectively. The magnitude of insulin secretion potentiation was shown at 1.56 fold (*P*<0.001), 1.30 fold (*P*<0.001), and 1.32 fold (*P*<0.001), when incubated together with 100 *μ*M IBMX, 300 *μ*M of diazoxide, and 30 mM of KCl, respectively, when compared to 500 *μ*g/ml* Ficus deltoidea *var.* motleyana* alone. The magnitude of insulin secretion inhibition was shown at 0.79 fold (*P*<0.001) when incubated with 200 *μ*M tolbutamide, when compared to 500 *μ*g/ml* Ficus deltoidea *var.* motleyana* alone.

## 4. Discussion

The mechanism underlying the insulin secretion has been well established [10]. However, the insulin secretion ability triggered by* Ficus deltoidea *varieties, which has been reported to possess antidiabetic properties [5, 6], remains underinvestigated. This study involves seven* Ficus deltoidea* varieties on cytotoxicity, insulin secretion, and elucidation of insulin secretion mechanism in BRIN BD 11 pancreatic beta cell.

A viability study on BRIN BD11 cell was performed to evaluate the toxicity of* Ficus deltoidea* methanolic extracts. In MTT assay, mitochondrial dehydrogenase enzyme, an indicator for healthy living cells, reduces tetrazolium salt to formazan crystals [7, 11, 12]. Therefore, from the viability assay carried out, only* Ficus deltoidea *var.* kunstleri *and* Ficus deltoidea *var.* intermedia *demonstrated toxicity at 500 and 1000 *μ*g/ml, while other extracts demonstrated a nontoxic effect towards BRIN BD11 cell. This result indicates that a concentration of 500 *μ*g/ml and above is toxic to BRIN BD11 cell for certain extracts [7]. The toxicity of the extract is due to the presence of phenolic compound that can potentially react with other compounds, thus inhibiting metabolic activities in the cell [12].

Insulin is an anabolic hormone that regulates glucose homeostasis [13]. Among the molecular mechanisms of action that relates to diabetes is the insulin secretion mechanism which occurs in the pancreatic beta cell. A lack of insulin secreted by the pancreatic beta cells can lead to hyperglycaemia. This is because blood glucose levels tend to increase, since glucose is not taken by muscle and adipose cells, thus interfering with glucose homeostasis. In general, insulin secretion triggered with glucose occurs through two pathways, namely, K_ATP_ dependent and independent pathways [14]. In the K_ATP_ dependent pathway, glucose that enters into the cell through glucose transporters will undergo glycolysis, where Adenosine Triphosphate (ATP) is formed, as the product will increase the cytoplasmic ATP and close the K_ATP_ dependent channel. The closure of the K_ATP_ dependent channel increases the membrane potential, causing membrane depolarization. From this, L type voltage dependent calcium channel (VDCC) opens and intrigues an influx of calcium ion into the cell. The increase of cytosolic Ca^2+^ concentration increases insulin exocytosis and eventually promotes insulin secretion. In addition, insulin secretion can also be manipulated through targeting the K_ATP_ dependent channel receptor. Previous work indicates that the closing of the K_ATP_ channel increases membrane depolarization, thus enhancing insulin secretion [9, 15–17]. In addition, the insulin concentration being secreted can be observed in a dose-dependent action when treated with* Ficus deltoidea *var.* deltoidea, angustifolia, *and* motleyana*, while the highest concentration of insulin being secreted can be observed at 500 *μ*g/ml for* Ficus deltoidea *var.* trengganuensis, kunstleri, *and* intermedia* and 100 *μ*g/ml for* Ficus deltoidea *var.* bilobata*. The difference in the results between extracts shows that the cytotoxicity of specific concentrations can affect the insulin secretion concentration [18].

The optimal concentration for each extract was chosen for elucidation of insulin secretion mechanism assay, considering the viability test results. Insulinotropic agonists such as isobutylmethylxanthine (IBMX) and tolbutamide were chosen for testing the effect of the drugs and extracts on the ability to induce insulin secretion through closing K_ATP_ channel. In addition, an insulinotropic antagonist, namely, diazoxide, was chosen to test the inhibitory effect of insulin secretion by the modulator and the extracts. Potassium chloride was chosen as the modulator depolarize plasma membrane, thus triggering insulin release [9, 15]. Based on the results, only* Ficus deltoidea *var.* intermedia* was involved in K_ATP_ dependent pathway due to the significant insulin secretion increase, coupled by the extract with tolbutamide. In addition, the coupling of the extract with diazoxide showed a significant result for the magnitude of inhibition in the insulin secretion. This is because tolbutamide works by closing the K_ATP_ channel through high affinity binding with SUR1/Kir6.2, a sulphonylurea receptor, which by closing the channel will open the voltage gated calcium channel, thus increasing insulin exocytosis [19]. Diazoxide works by opening the K_ATP_ channel, thus abolishing the insulin secretion effect of *β* pancreatic cell [20]. In addition, K_ATP_ independent pathway is involved in exocytosis of insulin through several pathways that utilize metabolite, protein, hormone, and lipid [21–24]. Therefore, in this study, intracellular cAMP became among the main metabolites for K_ATP_ independent channel pathway. Moreover,* Ficus deltoidea *var.* kunstleri, intermedia, deltoidea*,* bilobata,* and* motleyana *standardized extracts showed a significant magnitude of insulin secretion when combined with IBMX, which shows that the extracts follow K_ATP_ independent channel pathways supported by lower insulin secretion when combined with tolbutamide and an increase in insulin secretion when combined with KCl. Isobutyl methylxanthine (IBMX), a nonselective phosphodiesterase inhibitor, works by raising the intracellular cAMP, thus enhancing insulin secretion through protein kinase A pathway [25]. The attenuation of insulin secretion when incubated with tolbutamide showed noninvolvement of K_ATP_ channel, and previous work indicated that tolbutamide can attenuate insulin secretion in prolonged exposure [26]. Finally,* Ficus deltoidea *var.* trengganuensis* was involved in the K_ATP_ independent pathway, as only coincubation between the extract and 30 mM KCl had a significant effect of insulin secretion potentiation (P<0.05). This is because KCl, a nonnutrient secretagogue, enhances insulin secretion by depolarizing the plasma membrane at 30 mM, which then increases the influx of calcium ions [27]. As a suggestion for further work, other drugs such as verapamil can be used to treat together with* Ficus deltoidea *var.* trengganuensis* to further investigate association of the K_ATP_ independent pathway for this extract.

## 5. Conclusion

In conclusion, standardized methanolic extracts of* Ficus deltoidea *from the evaluated varieties have the effect to inflict insulin production from pancreatic beta cells. The insulin secretory activity of most of the varieties was through the K_ATP_ dependent pathway, except for var.* trengganuensis*, which was also through the K_ATP_ independent pathway. The insulinotropic activity of these* Ficus deltoidea* varieties suggests that they can potentially be developed as a new phytopharmaceutical agent for the management of diabetes mellitus potentially for escalation of insulin secretion from insulin producing cells.

Further research on the isolation and identification of compounds that are responsible for antidiabetic properties of the extracts can be carried out in order to further investigate their mechanistic functions in the pathways involved.

## Figures and Tables

**Table 1:** Cytotoxicity study on BRIN BD11 pancreatic beta cells.

*F. deltoidea* varieties	Cell viability (%)					
	Control	10 *μ*g/ml	50 *μ*g/ml	100 *μ*g/ml	500 *μ*g/ml	1000 *μ*g/ml
*Trengganuensis*	100	110.55 ± 2.09^*∗*^	118.97 ± 1.13^*∗∗*^	133.63 ± 0.92^*∗∗∗*^	161.69 ± 0.27^*∗∗∗*^	75.03 ± 5.31^*∗∗∗*^
*Kunstleri*	100	89.49 ± 4.87^*∗*^	98.93 ± 1.95	109.67 ± 5.37^*∗*^	10.74 ± 0.41^*∗∗∗*^	10.65 ± 1.03^*∗∗∗*^
*Intermedia*	100	90.90 ± 2.27	103.21 ± 7.20	111.42 ± 7.36	8.24 ± 0.75^*∗∗∗*^	8.71 ± 0.92^*∗∗∗*^
*Deltoidea*	100	107.60 ± 0.89^*∗*^	105.45 ± 3.29	106.13 ± 1.34	129.06 ± 0.04^*∗∗∗*^	55.13 ± 1.47^*∗∗∗*^
*Angustifolia*	100	125.78 ± 1.93^*∗∗∗*^	123.04 ± 0.44^*∗∗∗*^	131.78 ± 1.12^*∗∗∗*^	151.42 ± 0.08^*∗∗∗*^	103.21 ± 0.18
*Bilobata*	100	87.54 ± 3.94^*∗*^	85.27 ± 2.98^*∗∗*^	93.01 ± 3.66	115.56 ± 0.83^*∗∗*^	23.31 ± 1.55^*∗∗∗*^
*Motleyana*	100	95.17 ± 1.41	89.91 ± 0.36^*∗∗*^	73.68 ± 2.34^*∗∗∗*^	121.39 ± 0.96^*∗∗∗*^	103.92 ± 0.97

	Control	10 *μ*M	100 *μ*M	200 *μ*M	1000 *μ*M	2000 *μ*M

Glybenclamide	100	95.53 ± 0.34^*∗∗*^	103.71 ± 1.25^*∗∗*^	113.90 ± 0.24^*∗∗∗*^	12.66 ± 0.56^*∗∗∗*^	4.43 ± 0.81^*∗∗∗*^

*Notes*. Effect of cytotoxicity of standardized methanolic* Ficus deltoidea* varieties and glybenclamide on BRIN BD11 with incubation of 72 hours. Results are exhibited as mean ± SD (n=8) of percentage of cell viability. ^*∗*^*P*<0.05, ^*∗∗*^*P*<0.01, and ^*∗∗∗*^*P*<0.001 compared to control.

## Data Availability

The data used to support the findings of this study are available from the corresponding author upon request.
